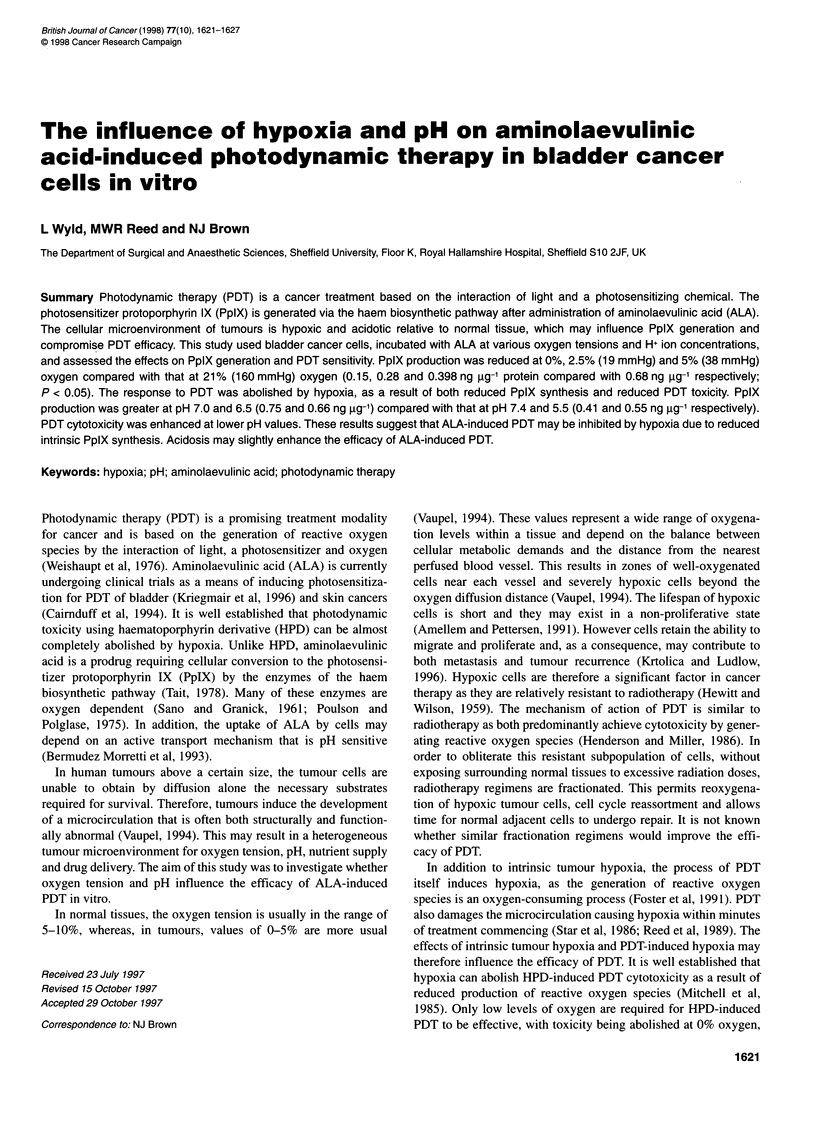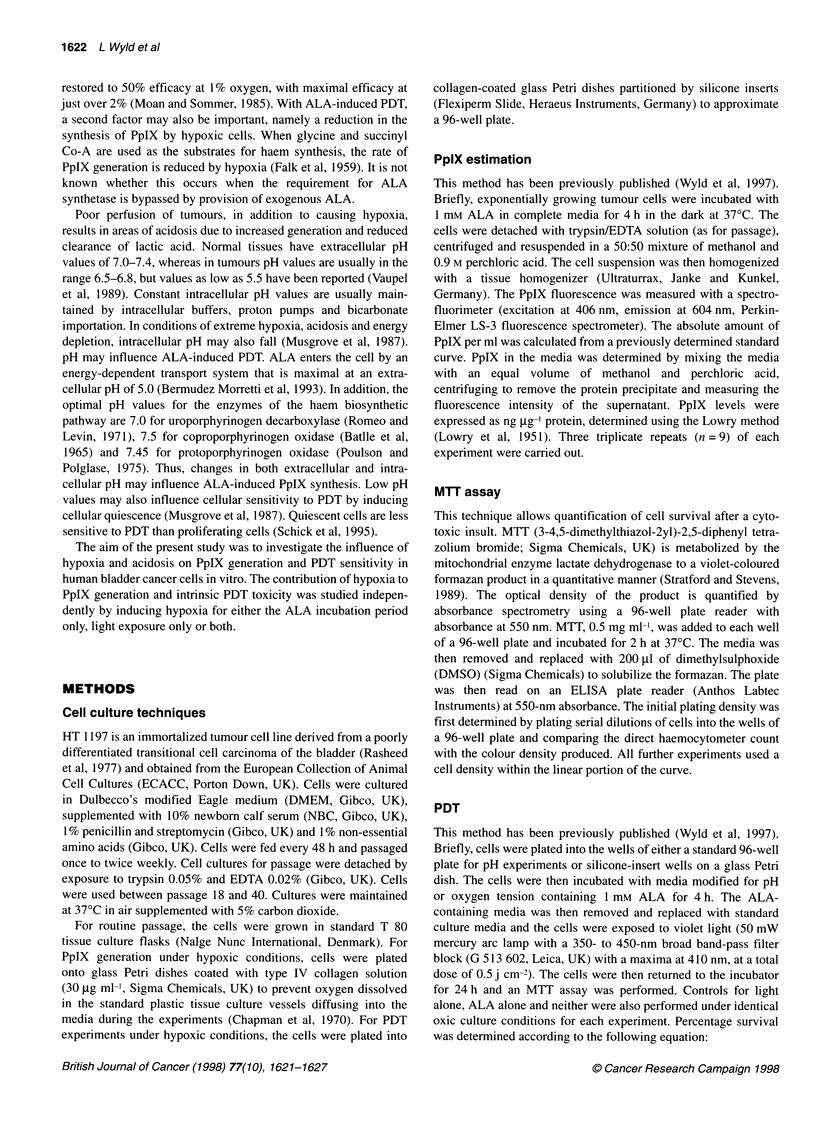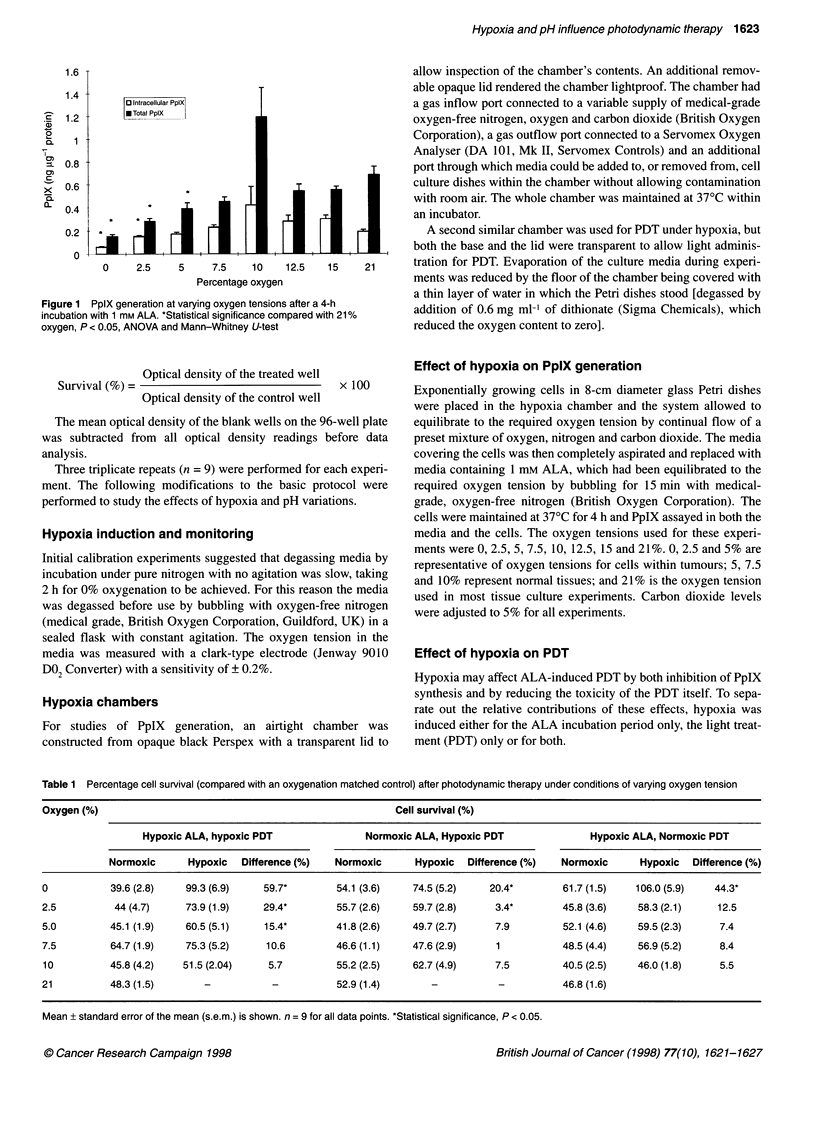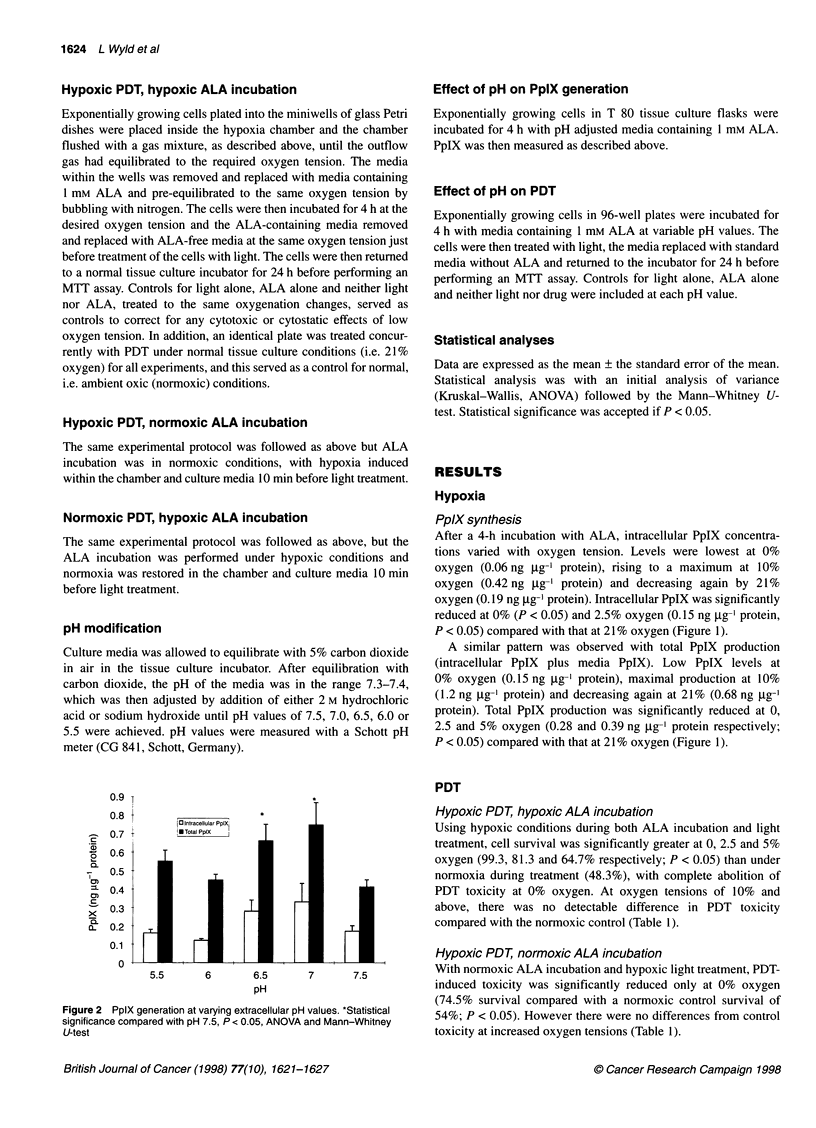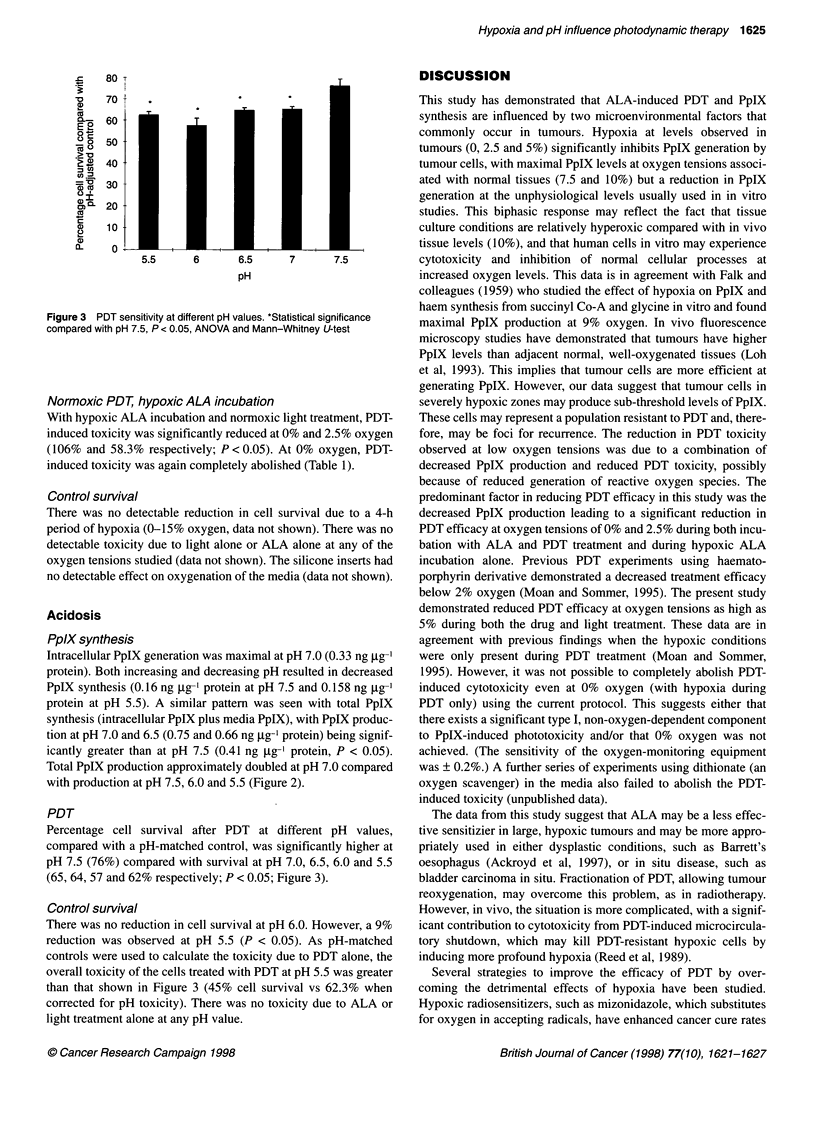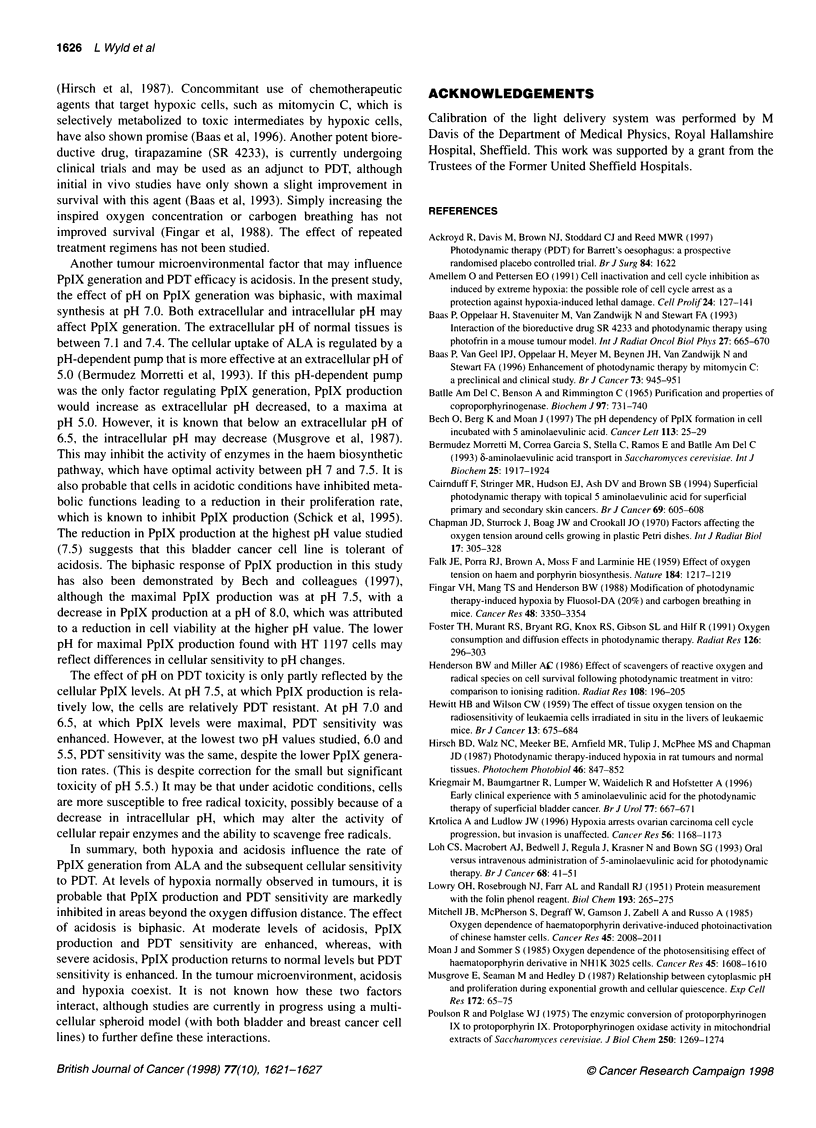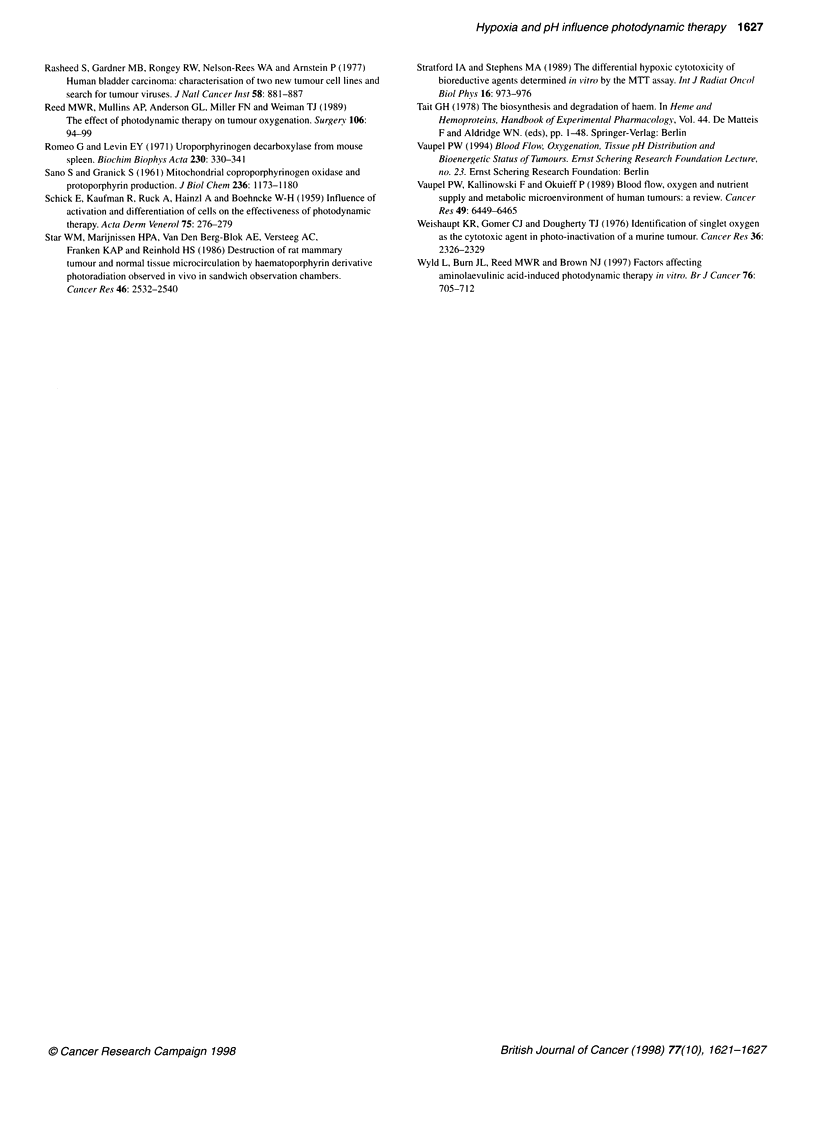# The influence of hypoxia and pH on aminolaevulinic acid-induced photodynamic therapy in bladder cancer cells in vitro.

**DOI:** 10.1038/bjc.1998.265

**Published:** 1998-05

**Authors:** L. Wyld, M. W. Reed, N. J. Brown

**Affiliations:** The Department of Surgical and Anaesthetic Sciences, Sheffield University, Royal Hallamshire Hospital, UK.

## Abstract

Photodynamic therapy (PDT) is a cancer treatment based on the interaction of light and a photosensitizing chemical. The photosensitizer protoporphyrin IX (PpIX) is generated via the haem biosynthetic pathway after administration of aminolaevulinic acid (ALA). The cellular microenvironment of tumours is hypoxic and acidotic relative to normal tissue, which may influence PpIX generation and compromise PDT efficacy. This study used bladder cancer cells, incubated with ALA at various oxygen tensions and H+ ion concentrations, and assessed the effects on PpIX generation and PDT sensitivity. PpIX production was reduced at 0%, 2.5% (19 mmHg) and 5% (38 mmHg) oxygen compared with that at 21% (160 mmHg) oxygen (0.15, 0.28 and 0.398 ng microg(-1) protein compared with 0.68 ng microg(-1) respectively; P < 0.05). The response to PDT was abolished by hypoxia, as a result of both reduced PpIX synthesis and reduced PDT toxicity. PpIX production was greater at pH 7.0 and 6.5 (0.75 and 0.66 ng microg(-1)) compared with that at pH 7.4 and 5.5 (0.41 and 0.55 ng microg(-1) respectively). PDT cytotoxicity was enhanced at lower pH values. These results suggest that ALA-induced PDT may be inhibited by hypoxia due to reduced intrinsic PpIX synthesis. Acidosis may slightly enhance the efficacy of ALA-induced PDT.


*British Journal of Cancer* (1998) **77**(10), 1621–1627
© 1998 Cancer Research Campaign

### The influence of hypoxia and pH on aminolaevulinic acid-induced photodynamic therapy in bladder cancer cells in vitro

**L Wyld, MWR Reed and NJ Brown**

The Department of Surgical and Anaesthetic Sciences, Sheffield University, Floor K, Royal Hallamshire Hospital, Sheffield S10 2JF, UK

**Summary** Photodynamic therapy (PDT) is a cancer treatment based on the interaction of light and a photosensitizing chemical. The photosensitizer protoporphyrin IX (PpIX) is generated via the haem biosynthetic pathway after administration of aminolaevulinic acid (ALA). The cellular microenvironment of tumours is hypoxic and acidotic relative to normal tissue, which may influence PpIX generation and compromise PDT efficacy. This study used bladder cancer cells, incubated with ALA at various oxygen tensions and $H^+$ ion concentrations, and assessed the effects on PpIX generation and PDT sensitivity. PpIX production was reduced at 0%, 2.5% (19 mmHg) and 5% (38 mmHg) oxygen compared with that at 21% (160 mmHg) oxygen (0.15, 0.28 and 0.398 ng $\mu g^{-1}$ protein compared with 0.68 ng $\mu g^{-1}$ respectively; $P < 0.05$). The response to PDT was abolished by hypoxia, as a result of both reduced PpIX synthesis and reduced PDT toxicity. PpIX production was greater at pH 7.0 and 6.5 (0.75 and 0.66 ng $\mu g^{-1}$) compared with that at pH 7.4 and 5.5 (0.41 and 0.55 ng $\mu g^{-1}$ respectively). PDT cytotoxicity was enhanced at lower pH values. These results suggest that ALA-induced PDT may be inhibited by hypoxia due to reduced intrinsic PpIX synthesis. Acidosis may slightly enhance the efficacy of ALA-induced PDT.

**Keywords:** hypoxia; pH; aminolaevulinic acid; photodynamic therapy

Photodynamic therapy (PDT) is a promising treatment modality for cancer and is based on the generation of reactive oxygen species by the interaction of light, a photosensitizer and oxygen (Weishaupt et al, 1976). Aminolaevulinic acid (ALA) is currently undergoing clinical trials as a means of inducing photosensitization for PDT of bladder (Kriegmair et al, 1996) and skin cancers (Cairnduff et al, 1994). It is well established that photodynamic toxicity using haematoporphyrin derivative (HPD) can be almost completely abolished by hypoxia. Unlike HPD, aminolaevulinic acid is a prodrug requiring cellular conversion to the photosensitizer protoporphyrin IX (PpIX) by the enzymes of the haem biosynthetic pathway (Tait, 1978). Many of these enzymes are oxygen dependent (Sano and Granick, 1961; Poulson and Polglase, 1975). In addition, the uptake of ALA by cells may depend on an active transport mechanism that is pH sensitive (Bermudez Morretti et al, 1993).

In human tumours above a certain size, the tumour cells are unable to obtain by diffusion alone the necessary substrates required for survival. Therefore, tumours induce the development of a microcirculation that is often both structurally and functionally abnormal (Vaupel, 1994). This may result in a heterogeneous tumour microenvironment for oxygen tension, pH, nutrient supply and drug delivery. The aim of this study was to investigate whether oxygen tension and pH influence the efficacy of ALA-induced PDT in vitro.

In normal tissues, the oxygen tension is usually in the range of 5–10%, whereas, in tumours, values of 0–5% are more usual (Vaupel, 1994). These values represent a wide range of oxygenation levels within a tissue and depend on the balance between cellular metabolic demands and the distance from the nearest perfused blood vessel. This results in zones of well-oxygenated cells near each vessel and severely hypoxic cells beyond the oxygen diffusion distance (Vaupel, 1994). The lifespan of hypoxic cells is short and they may exist in a non-proliferative state (Amellem and Pettersen, 1991). However cells retain the ability to migrate and proliferate and, as a consequence, may contribute to both metastasis and tumour recurrence (Krtolica and Ludlow, 1996). Hypoxic cells are therefore a significant factor in cancer therapy as they are relatively resistant to radiotherapy (Hewitt and Wilson, 1959). The mechanism of action of PDT is similar to radiotherapy as both predominantly achieve cytotoxicity by generating reactive oxygen species (Henderson and Miller, 1986). In order to obliterate this resistant subpopulation of cells, without exposing surrounding normal tissues to excessive radiation doses, radiotherapy regimens are fractionated. This permits reoxygenation of hypoxic tumour cells, cell cycle reassortment and allows time for normal adjacent cells to undergo repair. It is not known whether similar fractionation regimens would improve the efficacy of PDT.

In addition to intrinsic tumour hypoxia, the process of PDT itself induces hypoxia, as the generation of reactive oxygen species is an oxygen-consuming process (Foster et al, 1991). PDT also damages the microcirculation causing hypoxia within minutes of treatment commencing (Star et al, 1986; Reed et al, 1989). The effects of intrinsic tumour hypoxia and PDT-induced hypoxia may therefore influence the efficacy of PDT. It is well established that hypoxia can abolish HPD-induced PDT cytotoxicity as a result of reduced production of reactive oxygen species (Mitchell et al, 1985). Only low levels of oxygen are required for HPD-induced PDT to be effective, with toxicity being abolished at 0% oxygen,

*Received 23 July 1997*
*Revised 15 October 1997*
*Accepted 29 October 1997*

*Correspondence to:* NJ Brown restored to 50% efficacy at 1% oxygen, with maximal efficacy at just over 2% (Moan and Sommer, 1985). With ALA-induced PDT, a second factor may also be important, namely a reduction in the synthesis of PpIX by hypoxic cells. When glycine and succinyl Co-A are used as the substrates for haem synthesis, the rate of PpIX generation is reduced by hypoxia (Falk et al, 1959). It is not known whether this occurs when the requirement for ALA synthetase is bypassed by provision of exogenous ALA.

Poor perfusion of tumours, in addition to causing hypoxia, results in areas of acidosis due to increased generation and reduced clearance of lactic acid. Normal tissues have extracellular pH values of 7.0–7.4, whereas in tumours pH values are usually in the range 6.5–6.8, but values as low as 5.5 have been reported (Vaupel et al, 1989). Constant intracellular pH values are usually maintained by intracellular buffers, proton pumps and bicarbonate importation. In conditions of extreme hypoxia, acidosis and energy depletion, intracellular pH may also fall (Musgrove et al, 1987). pH may influence ALA-induced PDT. ALA enters the cell by an energy-dependent transport system that is maximal at an extracellular pH of 5.0 (Bermudez Morretti et al, 1993). In addition, the optimal pH values for the enzymes of the haem biosynthetic pathway are 7.0 for uroporphyrinogen decarboxylase (Romeo and Levin, 1971), 7.5 for coproporphyrinogen oxidase (Batlle et al, 1965) and 7.45 for protoporphyrinogen oxidase (Poulson and Polglase, 1975). Thus, changes in both extracellular and intracellular pH may influence ALA-induced PpIX synthesis. Low pH values may also influence cellular sensitivity to PDT by inducing cellular quiescence (Musgrove et al, 1987). Quiescent cells are less sensitive to PDT than proliferating cells (Schick et al, 1995).

The aim of the present study was to investigate the influence of hypoxia and acidosis on PpIX generation and PDT sensitivity in human bladder cancer cells in vitro. The contribution of hypoxia to PpIX generation and intrinsic PDT toxicity was studied independently by inducing hypoxia for either the ALA incubation period only, light exposure only or both.

### METHODS

### Cell culture techniques

HT 1197 is an immortalized tumour cell line derived from a poorly differentiated transitional cell carcinoma of the bladder (Rasheed et al, 1977) and obtained from the European Collection of Animal Cell Cultures (ECACC, Porton Down, UK). Cells were cultured in Dulbecco's modified Eagle medium (DMEM, Gibco, UK), supplemented with 10% newborn calf serum (NBC, Gibco, UK), 1% penicillin and streptomycin (Gibco, UK) and 1% non-essential amino acids (Gibco, UK). Cells were fed every 48 h and passaged once to twice weekly. Cell cultures for passage were detached by exposure to trypsin 0.05% and EDTA 0.02% (Gibco, UK). Cells were used between passage 18 and 40. Cultures were maintained at 37°C in air supplemented with 5% carbon dioxide.

For routine passage, the cells were grown in standard T 80 tissue culture flasks (Nalge Nunc International, Denmark). For PpIX generation under hypoxic conditions, cells were plated onto glass Petri dishes coated with type IV collagen solution (30 μg $ml^{-1}$, Sigma Chemicals, UK) to prevent oxygen dissolved in the standard plastic tissue culture vessels diffusing into the media during the experiments (Chapman et al, 1970). For PDT experiments under hypoxic conditions, the cells were plated into collagen-coated glass Petri dishes partitioned by silicone inserts (Flexiperm Slide, Heraeus Instruments, Germany) to approximate a 96-well plate.

### PpIX estimation

This method has been previously published (Wyld et al, 1997). Briefly, exponentially growing tumour cells were incubated with 1 mM ALA in complete media for 4 h in the dark at 37°C. The cells were detached with trypsin/EDTA solution (as for passage), centrifuged and resuspended in a 50:50 mixture of methanol and 0.9 M perchloric acid. The cell suspension was then homogenized with a tissue homogenizer (Ultraturrax, Janke and Kunkel, Germany). The PpIX fluorescence was measured with a spectrofluorimeter (excitation at 406 nm, emission at 604 nm, Perkin-Elmer LS-3 fluorescence spectrometer). The absolute amount of PpIX per ml was calculated from a previously determined standard curve. PpIX in the media was determined by mixing the media with an equal volume of methanol and perchloric acid, centrifuging to remove the protein precipitate and measuring the fluorescence intensity of the supernatant. PpIX levels were expressed as ng $\mu g^{-1}$ protein, determined using the Lowry method (Lowry et al, 1951). Three triplicate repeats ($n = 9$) of each experiment were carried out.

### MTT assay

This technique allows quantification of cell survival after a cytotoxic insult. MTT (3-4,5-dimethylthiazol-2yl)-2,5-diphenyl tetrazolium bromide; Sigma Chemicals, UK) is metabolized by the mitochondrial enzyme lactate dehydrogenase to a violet-coloured formazan product in a quantitative manner (Stratford and Stevens, 1989). The optical density of the product is quantified by absorbance spectrometry using a 96-well plate reader with absorbance at 550 nm. MTT, 0.5 mg $ml^{-1}$, was added to each well of a 96-well plate and incubated for 2 h at 37°C. The media was then removed and replaced with 200 μl of dimethylsulphoxide (DMSO) (Sigma Chemicals) to solubilize the formazan. The plate was then read on an ELISA plate reader (Anthos Labtec Instruments) at 550-nm absorbance. The initial plating density was first determined by plating serial dilutions of cells into the wells of a 96-well plate and comparing the direct haemocytometer count with the colour density produced. All further experiments used a cell density within the linear portion of the curve.

### PDT

This method has been previously published (Wyld et al, 1997). Briefly, cells were plated into the wells of either a standard 96-well plate for pH experiments or silicone-insert wells on a glass Petri dish. The cells were then incubated with media modified for pH or oxygen tension containing 1 mM ALA for 4 h. The ALA-containing media was then removed and replaced with standard culture media and the cells were exposed to violet light (50 mW mercury arc lamp with a 350- to 450-nm broad band-pass filter block (G 513 602, Leica, UK) with a maxima at 410 nm, at a total dose of 0.5 j $cm^{-2}$). The cells were then returned to the incubator for 24 h and an MTT assay was performed. Controls for light alone, ALA alone and neither were also performed under identical oxic culture conditions for each experiment. Percentage survival was determined according to the following equation:

 © Cancer Research Campaign 1998

**Figure 1** PpIX generation at varying oxygen tensions after a 4-h incubation with 1 mM ALA. *Statistical significance compared with 21% oxygen, $P < 0.05$, ANOVA and Mann–Whitney $U$-test

$$\text{Survival (\%)} = \frac{\text{Optical density of the treated well}}{\text{Optical density of the control well}} \times 100$$

The mean optical density of the blank wells on the 96-well plate was subtracted from all optical density readings before data analysis.

Three triplicate repeats ($n = 9$) were performed for each experiment. The following modifications to the basic protocol were performed to study the effects of hypoxia and pH variations.

### Hypoxia induction and monitoring

Initial calibration experiments suggested that degassing media by incubation under pure nitrogen with no agitation was slow, taking 2 h for 0% oxygenation to be achieved. For this reason the media was degassed before use by bubbling with oxygen-free nitrogen (medical grade, British Oxygen Corporation, Guildford, UK) in a sealed flask with constant agitation. The oxygen tension in the media was measured with a clark-type electrode (Jenway 9010 $DO_2$ Converter) with a sensitivity of ± 0.2%.

### Hypoxia chambers

For studies of PpIX generation, an airtight chamber was constructed from opaque black Perspex with a transparent lid to allow inspection of the chamber's contents. An additional removable opaque lid rendered the chamber lightproof. The chamber had a gas inflow port connected to a variable supply of medical-grade oxygen-free nitrogen, oxygen and carbon dioxide (British Oxygen Corporation), a gas outflow port connected to a Servomex Oxygen Analyser (DA 101, Mk II, Servomex Controls) and an additional port through which media could be added to, or removed from, cell culture dishes within the chamber without allowing contamination with room air. The whole chamber was maintained at 37°C within an incubator.

A second similar chamber was used for PDT under hypoxia, but both the base and the lid were transparent to allow light administration for PDT. Evaporation of the culture media during experiments was reduced by the floor of the chamber being covered with a thin layer of water in which the Petri dishes stood [degassed by addition of 0.6 mg $ml^{-1}$ of dithionate (Sigma Chemicals), which reduced the oxygen content to zero].

### Effect of hypoxia on PpIX generation

Exponentially growing cells in 8-cm diameter glass Petri dishes were placed in the hypoxia chamber and the system allowed to equilibrate to the required oxygen tension by continual flow of a preset mixture of oxygen, nitrogen and carbon dioxide. The media covering the cells was then completely aspirated and replaced with media containing 1 mM ALA, which had been equilibrated to the required oxygen tension by bubbling for 15 min with medical-grade, oxygen-free nitrogen (British Oxygen Corporation). The cells were maintained at 37°C for 4 h and PpIX assayed in both the media and the cells. The oxygen tensions used for these experiments were 0, 2.5, 5, 7.5, 10, 12.5, 15 and 21%. 0, 2.5 and 5% are representative of oxygen tensions for cells within tumours; 5, 7.5 and 10% represent normal tissues; and 21% is the oxygen tension used in most tissue culture experiments. Carbon dioxide levels were adjusted to 5% for all experiments.

### Effect of hypoxia on PDT

Hypoxia may affect ALA-induced PDT by both inhibition of PpIX synthesis and by reducing the toxicity of the PDT itself. To separate out the relative contributions of these effects, hypoxia was induced either for the ALA incubation period only, the light treatment (PDT) only or for both.

**Table 1** Percentage cell survival (compared with an oxygenation matched control) after photodynamic therapy under conditions of varying oxygen tension

| Oxygen (%) | Cell survival (%) | | | | | | | | |
|---|---|---|---|---|---|---|---|---|---|
| | Hypoxic ALA, hypoxic PDT | | | Normoxic ALA, Hypoxic PDT | | | Hypoxic ALA, Normoxic PDT | | |
| | Normoxic | Hypoxic | Difference (%) | Normoxic | Hypoxic | Difference (%) | Normoxic | Hypoxic | Difference (%) |
| 0 | 39.6 (2.8) | 99.3 (6.9) | 59.7* | 54.1 (3.6) | 74.5 (5.2) | 20.4* | 61.7 (1.5) | 106.0 (5.9) | 44.3* |
| 2.5 | 44 (4.7) | 73.9 (1.9) | 29.4* | 55.7 (2.6) | 59.7 (2.8) | 3.4* | 45.8 (3.6) | 58.3 (2.1) | 12.5 |
| 5.0 | 45.1 (1.9) | 60.5 (5.1) | 15.4* | 41.8 (2.6) | 49.7 (2.7) | 7.9 | 52.1 (4.6) | 59.5 (2.3) | 7.4 |
| 7.5 | 64.7 (1.9) | 75.3 (5.2) | 10.6 | 46.6 (1.1) | 47.6 (2.9) | 1 | 48.5 (4.4) | 56.9 (5.2) | 8.4 |
| 10 | 45.8 (4.2) | 51.5 (2.04) | 5.7 | 55.2 (2.5) | 62.7 (4.9) | 7.5 | 40.5 (2.5) | 46.0 (1.8) | 5.5 |
| 21 | 48.3 (1.5) | – | – | 52.9 (1.4) | – | – | 46.8 (1.6) | | |

Mean ± standard error of the mean (s.e.m.) is shown. $n = 9$ for all data points. *Statistical significance, $P < 0.05$.

© Cancer Research Campaign 1998

### Hypoxic PDT, hypoxic ALA incubation

Exponentially growing cells plated into the miniwells of glass Petri dishes were placed inside the hypoxia chamber and the chamber flushed with a gas mixture, as described above, until the outflow gas had equilibrated to the required oxygen tension. The media within the wells was removed and replaced with media containing 1 mM ALA and pre-equilibrated to the same oxygen tension by bubbling with nitrogen. The cells were then incubated for 4 h at the desired oxygen tension and the ALA-containing media removed and replaced with ALA-free media at the same oxygen tension just before treatment of the cells with light. The cells were then returned to a normal tissue culture incubator for 24 h before performing an MTT assay. Controls for light alone, ALA alone and neither light nor ALA, treated to the same oxygenation changes, served as controls to correct for any cytotoxic or cytostatic effects of low oxygen tension. In addition, an identical plate was treated concurrently with PDT under normal tissue culture conditions (i.e. 21% oxygen) for all experiments, and this served as a control for normal, i.e. ambient oxic (normoxic) conditions.

### Hypoxic PDT, normoxic ALA incubation

The same experimental protocol was followed as above but ALA incubation was in normoxic conditions, with hypoxia induced within the chamber and culture media 10 min before light treatment.

### Normoxic PDT, hypoxic ALA incubation

The same experimental protocol was followed as above, but the ALA incubation was performed under hypoxic conditions and normoxia was restored in the chamber and culture media 10 min before light treatment.

### pH modification

Culture media was allowed to equilibrate with 5% carbon dioxide in air in the tissue culture incubator. After equilibration with carbon dioxide, the pH of the media was in the range 7.3–7.4, which was then adjusted by addition of either 2 M hydrochloric acid or sodium hydroxide until pH values of 7.5, 7.0, 6.5, 6.0 or 5.5 were achieved. pH values were measured with a Schott pH meter (CG 841, Schott, Germany).

**Figure 2** PpIX generation at varying extracellular pH values. *Statistical significance compared with pH 7.5, $P < 0.05$, ANOVA and Mann–Whitney *U*-test

### Effect of pH on PpIX generation

Exponentially growing cells in T 80 tissue culture flasks were incubated for 4 h with pH adjusted media containing 1 mM ALA. PpIX was then measured as described above.

### Effect of pH on PDT

Exponentially growing cells in 96-well plates were incubated for 4 h with media containing 1 mM ALA at variable pH values. The cells were then treated with light, the media replaced with standard media without ALA and returned to the incubator for 24 h before performing an MTT assay. Controls for light alone, ALA alone and neither light nor drug were included at each pH value.

### Statistical analyses

Data are expressed as the mean ± the standard error of the mean. Statistical analysis was with an initial analysis of variance (Kruskal–Wallis, ANOVA) followed by the Mann–Whitney *U*-test. Statistical significance was accepted if $P < 0.05$.

### RESULTS

### Hypoxia

### *PpIX synthesis*

After a 4-h incubation with ALA, intracellular PpIX concentrations varied with oxygen tension. Levels were lowest at 0% oxygen (0.06 ng $\mu g^{-1}$ protein), rising to a maximum at 10% oxygen (0.42 ng $\mu g^{-1}$ protein) and decreasing again by 21% oxygen (0.19 ng $\mu g^{-1}$ protein). Intracellular PpIX was significantly reduced at 0% ($P < 0.05$) and 2.5% oxygen (0.15 ng $\mu g^{-1}$ protein, $P < 0.05$) compared with that at 21% oxygen (Figure 1).

A similar pattern was observed with total PpIX production (intracellular PpIX plus media PpIX). Low PpIX levels at 0% oxygen (0.15 ng $\mu g^{-1}$ protein), maximal production at 10% (1.2 ng $\mu g^{-1}$ protein) and decreasing again at 21% (0.68 ng $\mu g^{-1}$ protein). Total PpIX production was significantly reduced at 0, 2.5 and 5% oxygen (0.28 and 0.39 ng $\mu g^{-1}$ protein respectively; $P < 0.05$) compared with that at 21% oxygen (Figure 1).

### PDT

### *Hypoxic PDT, hypoxic ALA incubation*

Using hypoxic conditions during both ALA incubation and light treatment, cell survival was significantly greater at 0, 2.5 and 5% oxygen (99.3, 81.3 and 64.7% respectively; $P < 0.05$) than under normoxia during treatment (48.3%), with complete abolition of PDT toxicity at 0% oxygen. At oxygen tensions of 10% and above, there was no detectable difference in PDT toxicity compared with the normoxic control (Table 1).

### *Hypoxic PDT, normoxic ALA incubation*

With normoxic ALA incubation and hypoxic light treatment, PDT-induced toxicity was significantly reduced only at 0% oxygen (74.5% survival compared with a normoxic control survival of 54%; $P < 0.05$). However there were no differences from control toxicity at increased oxygen tensions (Table 1).

© Cancer Research Campaign 1998

**Figure 3** PDT sensitivity at different pH values. *Statistical significance compared with pH 7.5, $P < 0.05$, ANOVA and Mann–Whitney *U*-test

### *Normoxic PDT, hypoxic ALA incubation*

With hypoxic ALA incubation and normoxic light treatment, PDT-induced toxicity was significantly reduced at 0% and 2.5% oxygen (106% and 58.3% respectively; $P < 0.05$). At 0% oxygen, PDT-induced toxicity was again completely abolished (Table 1).

### *Control survival*

There was no detectable reduction in cell survival due to a 4-h period of hypoxia (0–15% oxygen, data not shown). There was no detectable toxicity due to light alone or ALA alone at any of the oxygen tensions studied (data not shown). The silicone inserts had no detectable effect on oxygenation of the media (data not shown).

### Acidosis

### *PpIX synthesis*

Intracellular PpIX generation was maximal at pH 7.0 (0.33 ng $\mu g^{-1}$ protein). Both increasing and decreasing pH resulted in decreased PpIX synthesis (0.16 ng $\mu g^{-1}$ protein at pH 7.5 and 0.158 ng $\mu g^{-1}$ protein at pH 5.5). A similar pattern was seen with total PpIX synthesis (intracellular PpIX plus media PpIX), with PpIX production at pH 7.0 and 6.5 (0.75 and 0.66 ng $\mu g^{-1}$ protein) being significantly greater than at pH 7.5 (0.41 ng $\mu g^{-1}$ protein, $P < 0.05$). Total PpIX production approximately doubled at pH 7.0 compared with production at pH 7.5, 6.0 and 5.5 (Figure 2).

### *PDT*

Percentage cell survival after PDT at different pH values, compared with a pH-matched control, was significantly higher at pH 7.5 (76%) compared with survival at pH 7.0, 6.5, 6.0 and 5.5 (65, 64, 57 and 62% respectively; $P < 0.05$; Figure 3).

### *Control survival*

There was no reduction in cell survival at pH 6.0. However, a 9% reduction was observed at pH 5.5 ($P < 0.05$). As pH-matched controls were used to calculate the toxicity due to PDT alone, the overall toxicity of the cells treated with PDT at pH 5.5 was greater than that shown in Figure 3 (45% cell survival vs 62.3% when corrected for pH toxicity). There was no toxicity due to ALA or light treatment alone at any pH value.

### DISCUSSION

This study has demonstrated that ALA-induced PDT and PpIX synthesis are influenced by two microenvironmental factors that commonly occur in tumours. Hypoxia at levels observed in tumours (0, 2.5 and 5%) significantly inhibits PpIX generation by tumour cells, with maximal PpIX levels at oxygen tensions associated with normal tissues (7.5 and 10%) but a reduction in PpIX generation at the unphysiological levels usually used in in vitro studies. This biphasic response may reflect the fact that tissue culture conditions are relatively hyperoxic compared with in vivo tissue levels (10%), and that human cells in vitro may experience cytotoxicity and inhibition of normal cellular processes at increased oxygen levels. This data is in agreement with Falk and colleagues (1959) who studied the effect of hypoxia on PpIX and haem synthesis from succinyl Co-A and glycine in vitro and found maximal PpIX production at 9% oxygen. In vivo fluorescence microscopy studies have demonstrated that tumours have higher PpIX levels than adjacent normal, well-oxygenated tissues (Loh et al, 1993). This implies that tumour cells are more efficient at generating PpIX. However, our data suggest that tumour cells in severely hypoxic zones may produce sub-threshold levels of PpIX. These cells may represent a population resistant to PDT and, therefore, may be foci for recurrence. The reduction in PDT toxicity observed at low oxygen tensions was due to a combination of decreased PpIX production and reduced PDT toxicity, possibly because of reduced generation of reactive oxygen species. The predominant factor in reducing PDT efficacy in this study was the decreased PpIX production leading to a significant reduction in PDT efficacy at oxygen tensions of 0% and 2.5% during both incubation with ALA and PDT treatment and during hypoxic ALA incubation alone. Previous PDT experiments using haematoporphyrin derivative demonstrated a decreased treatment efficacy below 2% oxygen (Moan and Sommer, 1995). The present study demonstrated reduced PDT efficacy at oxygen tensions as high as 5% during both the drug and light treatment. These data are in agreement with previous findings when the hypoxic conditions were only present during PDT treatment (Moan and Sommer, 1995). However, it was not possible to completely abolish PDT-induced cytotoxicity even at 0% oxygen (with hypoxia during PDT only) using the current protocol. This suggests either that there exists a significant type I, non-oxygen-dependent component to PpIX-induced phototoxicity and/or that 0% oxygen was not achieved. (The sensitivity of the oxygen-monitoring equipment was ± 0.2%.) A further series of experiments using dithionate (an oxygen scavenger) in the media also failed to abolish the PDT-induced toxicity (unpublished data).

The data from this study suggest that ALA may be a less effective sensitizier in large, hypoxic tumours and may be more appropriately used in either dysplastic conditions, such as Barrett's oesophagus (Ackroyd et al, 1997), or in situ disease, such as bladder carcinoma in situ. Fractionation of PDT, allowing tumour reoxygenation, may overcome this problem, as in radiotherapy. However, in vivo, the situation is more complicated, with a significant contribution to cytotoxicity from PDT-induced microcirculatory shutdown, which may kill PDT-resistant hypoxic cells by inducing more profound hypoxia (Reed et al, 1989).

Several strategies to improve the efficacy of PDT by overcoming the detrimental effects of hypoxia have been studied. Hypoxic radiosensitizers, such as mizonidazole, which substitutes for oxygen in accepting radicals, have enhanced cancer cure rates

© Cancer Research Campaign 1998

(Hirsch et al, 1987). Concommitant use of chemotherapeutic agents that target hypoxic cells, such as mitomycin C, which is selectively metabolized to toxic intermediates by hypoxic cells, have also shown promise (Baas et al, 1996). Another potent bioreductive drug, tirapazamine (SR 4233), is currently undergoing clinical trials and may be used as an adjunct to PDT, although initial in vivo studies have only shown a slight improvement in survival with this agent (Baas et al, 1993). Simply increasing the inspired oxygen concentration or carbogen breathing has not improved survival (Fingar et al, 1988). The effect of repeated treatment regimens has not been studied.

Another tumour microenvironmental factor that may influence PpIX generation and PDT efficacy is acidosis. In the present study, the effect of pH on PpIX generation was biphasic, with maximal synthesis at pH 7.0. Both extracellular and intracellular pH may affect PpIX generation. The extracellular pH of normal tissues is between 7.1 and 7.4. The cellular uptake of ALA is regulated by a pH-dependent pump that is more effective at an extracellular pH of 5.0 (Bermudez Morretti et al, 1993). If this pH-dependent pump was the only factor regulating PpIX generation, PpIX production would increase as extracellular pH decreased, to a maxima at pH 5.0. However, it is known that below an extracellular pH of 6.5, the intracellular pH may decrease (Musgrove et al, 1987). This may inhibit the activity of enzymes in the haem biosynthetic pathway, which have optimal activity between pH 7 and 7.5. It is also probable that cells in acidotic conditions have inhibited metabolic functions leading to a reduction in their proliferation rate, which is known to inhibit PpIX production (Schick et al, 1995). The reduction in PpIX production at the highest pH value studied (7.5) suggests that this bladder cancer cell line is tolerant of acidosis. The biphasic response of PpIX production in this study has also been demonstrated by Bech and colleagues (1997), although the maximal PpIX production was at pH 7.5, with a decrease in PpIX production at a pH of 8.0, which was attributed to a reduction in cell viability at the higher pH value. The lower pH for maximal PpIX production found with HT 1197 cells may reflect differences in cellular sensitivity to pH changes.

The effect of pH on PDT toxicity is only partly reflected by the cellular PpIX levels. At pH 7.5, at which PpIX production is relatively low, the cells are relatively PDT resistant. At pH 7.0 and 6.5, at which PpIX levels were maximal, PDT sensitivity was enhanced. However, at the lowest two pH values studied, 6.0 and 5.5, PDT sensitivity was the same, despite the lower PpIX generation rates. (This is despite correction for the small but significant toxicity of pH 5.5.) It may be that under acidotic conditions, cells are more susceptible to free radical toxicity, possibly because of a decrease in intracellular pH, which may alter the activity of cellular repair enzymes and the ability to scavenge free radicals.

In summary, both hypoxia and acidosis influence the rate of PpIX generation from ALA and the subsequent cellular sensitivity to PDT. At levels of hypoxia normally observed in tumours, it is probable that PpIX production and PDT sensitivity are markedly inhibited in areas beyond the oxygen diffusion distance. The effect of acidosis is biphasic. At moderate levels of acidosis, PpIX production and PDT sensitivity are enhanced, whereas, with severe acidosis, PpIX production returns to normal levels but PDT sensitivity is enhanced. In the tumour microenvironment, acidosis and hypoxia coexist. It is not known how these two factors interact, although studies are currently in progress using a multicellular spheroid model (with both bladder and breast cancer cell lines) to further define these interactions.

### ACKNOWLEDGEMENTS

Calibration of the light delivery system was performed by M Davis of the Department of Medical Physics, Royal Hallamshire Hospital, Sheffield. This work was supported by a grant from the Trustees of the Former United Sheffield Hospitals.

### REFERENCES

Ackroyd R, Davis M, Brown NJ, Stoddard CJ and Reed MWR (1997) Photodynamic therapy (PDT) for Barrett's oesophagus: a prospective randomised placebo controlled trial. *Br J Surg* **84**: 1622

Amellem O and Pettersen EO (1991) Cell inactivation and cell cycle inhibition as induced by extreme hypoxia: the possible role of cell cycle arrest as a protection against hypoxia-induced lethal damage. *Cell Prolif* **24**: 127–141

Baas P, Oppelaar H, Stavenuiter M, Van Zandwijk N and Stewart FA (1993) Interaction of the bioreductive drug SR 4233 and photodynamic therapy using photofrin in a mouse tumour model. *Int J Radiat Oncol Biol Phys* **27**: 665–670

Baas P, Van Geel IPJ, Oppelaar H, Meyer M, Beynen JH, Van Zandwijk N and Stewart FA (1996) Enhancement of photodynamic therapy by mitomycin C: a preclinical and clinical study. *Br J Cancer* **73**: 945–951

Batlle Am Del C, Benson A and Rimmington C (1965) Purification and properties of coproporphyrinogenase. *Biochem J* **97**: 731–740

Bech O, Berg K and Moan J (1997) The pH dependency of PpIX formation in cell incubated with 5 aminolaevulinic acid. *Cancer Lett* **113**: 25–29

Bermudez Morretti M, Correa Garcia S, Stella C, Ramos E and Batlle Am Del C (1993) δ-aminolaevulinic acid transport in *Saccharomyces cerevisiae*. *Int J Biochem* **25**: 1917–1924

Cairnduff F, Stringer MR, Hudson EJ, Ash DV and Brown SB (1994) Superficial photodynamic therapy with topical 5 aminolaevulinic acid for superficial primary and secondary skin cancers. *Br J Cancer* **69**: 605–608

Chapman JD, Sturrock J, Boag JW and Crookall JO (1970) Factors affecting the oxygen tension around cells growing in plastic Petri dishes. *Int J Radiat Biol* **17**: 305–328

Falk JE, Porra RJ, Brown A, Moss F and Larminie HE (1959) Effect of oxygen tension on haem and porphyrin biosynthesis. *Nature* **184**: 1217–1219

Fingar VH, Mang TS and Henderson BW (1988) Modification of photodynamic therapy-induced hypoxia by Fluosol-DA (20%) and carbogen breathing in mice. *Cancer Res* **48**: 3350–3354

Foster TH, Murant RS, Bryant RG, Knox RS, Gibson SL and Hilf R (1991) Oxygen consumption and diffusion effects in photodynamic therapy. *Radiat Res* **126**: 296–303

Henderson BW and Miller AC (1986) Effect of scavengers of reactive oxygen and radical species on cell survival following photodynamic treatment in vitro: comparison to ionising radition. *Radiat Res* **108**: 196–205

Hewitt HB and Wilson CW (1959) The effect of tissue oxygen tension on the radiosensitivity of leukaemia cells irradiated in situ in the livers of leukaemic mice. *Br J Cancer* **13**: 675–684

Hirsch BD, Walz NC, Meeker BE, Arnfield MR, Tulip J, McPhee MS and Chapman JD (1987) Photodynamic therapy-induced hypoxia in rat tumours and normal tissues. *Photochem Photobiol* **46**: 847–852

Kriegmair M, Baumgartner R, Lumper W, Waidelich R and Hofstetter A (1996) Early clinical experience with 5 aminolaevulinic acid for the photodynamic therapy of superficial bladder cancer. *Br J Urol* **77**: 667–671

Krtolica A and Ludlow JW (1996) Hypoxia arrests ovarian carcinoma cell cycle progression, but invasion is unaffected. *Cancer Res* **56**: 1168–1173

Loh CS, Macrobert AJ, Bedwell J, Regula J, Krasner N and Bown SG (1993) Oral versus intravenous administration of 5-aminolaevulinic acid for photodynamic therapy. *Br J Cancer* **68**: 41–51

Lowry OH, Rosebrough NJ, Farr AL and Randall RJ (1951) Protein measurement with the folin phenol reagent. *Biol Chem* **193**: 265–275

Mitchell JB, McPherson S, Degraff W, Gamson J, Zabell A and Russo A (1985) Oxygen dependence of haematoporphyrin derivative-induced photoinactivation of chinese hamster cells. *Cancer Res* **45**: 2008–2011

Moan J and Sommer S (1985) Oxygen dependence of the photosensitising effect of haematoporphyrin derivative in NH1K 3025 cells. *Cancer Res* **45**: 1608–1610

Musgrove E, Seaman M and Hedley D (1987) Relationship between cytoplasmic pH and proliferation during exponential growth and cellular quiescence. *Exp Cell Res* **172**: 65–75

Poulson R and Polglase WJ (1975) The enzymic conversion of protoporphyrinogen IX to protoporphyrin IX. Protoporphyrinogen oxidase activity in mitochondrial extracts of *Saccharomyces cerevisiae*. *J Biol Chem* **250**: 1269–1274

© Cancer Research Campaign 1998

Rasheed S, Gardner MB, Rongey RW, Nelson-Rees WA and Arnstein P (1977) Human bladder carcinoma: characterisation of two new tumour cell lines and search for tumour viruses. *J Natl Cancer Inst* **58**: 881–887

Reed MWR, Mullins AP, Anderson GL, Miller FN and Weiman TJ (1989) The effect of photodynamic therapy on tumour oxygenation. *Surgery* **106**: 94–99

Romeo G and Levin EY (1971) Uroporphyrinogen decarboxylase from mouse spleen. *Biochim Biophys Acta* **230**: 330–341

Sano S and Granick S (1961) Mitochondrial coproporphyrinogen oxidase and protoporphyrin production. *J Biol Chem* **236**: 1173–1180

Schick E, Kaufman R, Ruck A, Hainzl A and Boehncke W-H (1959) Influence of activation and differentiation of cells on the effectiveness of photodynamic therapy. *Acta Derm Venerol* **75**: 276–279

Star WM, Marijnissen HPA, Van Den Berg-Blok AE, Versteeg AC, Franken KAP and Reinhold HS (1986) Destruction of rat mammary tumour and normal tissue microcirculation by haematoporphyrin derivative photoradiation observed in vivo in sandwich observation chambers. *Cancer Res* **46**: 2532–2540

Stratford IA and Stephens MA (1989) The differential hypoxic cytotoxicity of bioreductive agents determined *in vitro* by the MTT assay. *Int J Radiat Oncol Biol Phys* **16**: 973–976

Tait GH (1978) The biosynthesis and degradation of haem. In *Heme and Hemoproteins, Handbook of Experimental Pharmacology*, Vol. 44. De Matteis F and Aldridge WN. (eds), pp. 1–48. Springer-Verlag: Berlin

Vaupel PW (1994) *Blood Flow, Oxygenation, Tissue pH Distribution and Bioenergetic Status of Tumours. Ernst Schering Research Foundation Lecture, no. 23.* Ernst Schering Research Foundation: Berlin

Vaupel PW, Kallinowski F and Okuieff P (1989) Blood flow, oxygen and nutrient supply and metabolic microenvironment of human tumours: a review. *Cancer Res* **49**: 6449–6465

Weishaupt KR, Gomer CJ and Dougherty TJ (1976) Identification of singlet oxygen as the cytotoxic agent in photo-inactivation of a murine tumour. *Cancer Res* **36**: 2326–2329

Wyld L, Burn JL, Reed MWR and Brown NJ (1997) Factors affecting aminolaevulinic acid-induced photodynamic therapy *in vitro*. *Br J Cancer* **76**: 705–712

© Cancer Research Campaign 1998